# Supplementary material for: Programmable de novo designed coiled coil-mediated phase separation in mammalian cells
Source: Nat Commun. 2023 Dec 2;14:7973. doi: 10.1038/s41467-023-43742-w (PMC10693550; doi:10.1038/s41467-023-43742-w)
Supplement: Supplementary file 1 — Supplementary Information [file 41467_2023_43742_MOESM1_ESM.pdf]

Programmable *de novo* designed coiled coil-mediated phase separation in mammalian cells

Supplementary Table 1: Amino acid sequences of the de novo designed CC set, used in the study

	Peptide name:	Color code	Amino acid sequence: gabcdef gabcdef gabcdef	Hydrophobic pattern	Electrostatic pattern
S1-S2	**S1**		KIQSLKE EISQLEQ KNSQLKE	IIN	KKEEKK
	**S2**		EIQSLEE KISQLKQ ENSQLEE	IIN	EEKKEE
S3-S4	**S3**		EIQSLEE ENSQLEQ KISQLKE	INI	EEEEKK
	**S4**		KIQSLKE KNSQLKQ EISQLEE	INI	KKKKEE
S1h-S2h	**S1h**		KIAALKE EIAALEQ KNAALKE	IIN	KKEEKK
	**S2h**		EIAALEE KIAALKQ ENAALEE	IIN	EEKKEE
S3h-S3h	**S3h**		EIAALEE ENAALEQ KIAALKE	INI	EEEEKK
	**S4h**		KIAALKE KNAALKQ EIAALEE	INI	KKKKEE
P5f-P6f	**P5f**		ENAALEE KIAALKE KNAALKE EIAALEA	NINI	EEKKKKEE
	**P6f**		KNAALKE EIAALEE ENAALEE KIAALKA	NINI	KKEEEEKK
P13f-P14f	**P13f**		KIAALKE EIAALEQ KNAALKE ENAALEA	IINN	KKEEKKEE
	**P14f**		EIAALEE KIAALKQ ENAALEE KNAALKA	IINN	EEKKEEKK
D1h-D2h	**D1h**		EIAALEQ KNAALKE	IN	EEKK
	**D2h**		KIAALKQ ENAALEE	IN	KKEE
D3h-D4h	**D3h**		ENAALEQ KIAALKE	NI	EEKK
	**D4h**		KNAALKQ EIAALEE	NI	KKEE
AP2-AP2	**APh2**		ELAAIEE QLAALAA KAEARKE		EE--KK
				Antiparallel homodimer	
GCN-GCN	**GCNh2**		KIAALEA KNAALEA EIAALKA	INI	KEKEEK
				Parallel homodimer	

Supplementary Table 2: CC-LLPS proteins used in the study and their molecular weights.

Location	Name:		*MW* (kDa):
Fig S3	mCit(S1)6-SAH	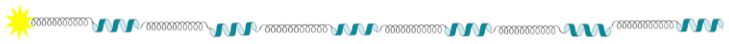	69.7
Fig S3	TagBFP(S2)6-gs	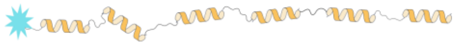	44.7
Fig S3	mCit(S3)6-SAH	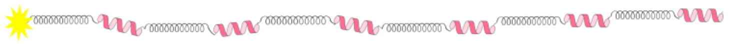	69.7
Fig S3	TagBFP(S4)6-gs	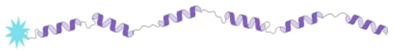	44.7
Fig 1, 2, S3, S4, S5, S14	mCit(S1-S3)3-SAH	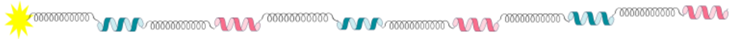	69.7
Fig 1, 2, S3, S4, S5	TagBFP(S2)3(S4)3-gs	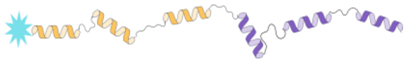	44.7
Fig 2h, S4	TagBFP(S2)3(S4)3-SAH	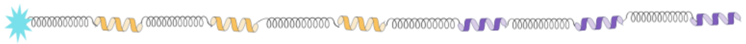	69.0
Fig 2g, S4	mCit(S1-S3)3-gs	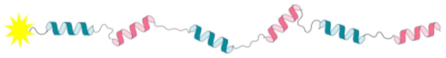	45.4
Fig 2a	mCit(S1-S3)2-SAH	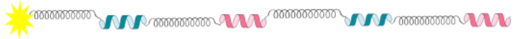	54.8
Fig 2a	TagBFP(S2)2(S4)2-gs	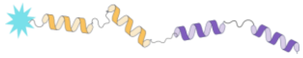	38.6
Fig 2a	mCit(S1-S3)1-SAH	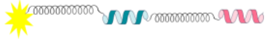	55.5
Fig 2a	TagBFP(S2-S4)1-gs	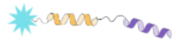	32.4
Fig S3	mCit(S1)3(S3)3-SAH	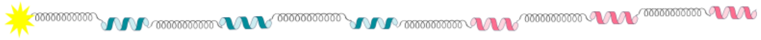	41.2
Fig S3, S4	TagBFP(S2-S4)3-gs	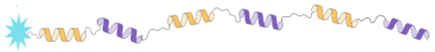	69.7
Fig 3, S6, S10, S15	mCit(S1h-S3h)3-gs	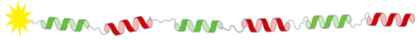	43.8
Fig 3, 5, S6, S10, S13, S15	TagBFP(S2h)3(S4h)3-gs	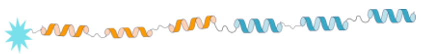	43.1
Fig 5, S13	(S1h-S3h)3-gs	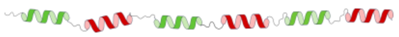	16.9
Fig 3, S6, S10	mCit(S1h)3(S3h)3-gs	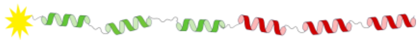	43.8
Fig 3, 5 S6, S10, S15	mCit(P5f-P13f)3-gs	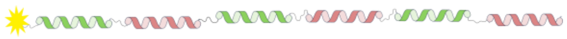	47.9
Fig 3, 5, S6, S10, S15	TagBFP(P6f)3(P14f)3-gs	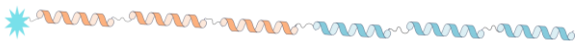	47.2
Fig 5, S13	(P6f)3(P14f)3-gs	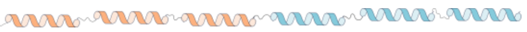	20.9
Fig 5	TagBFP(S2h)3(S4h)3-gs-P6f	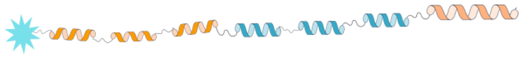	46.6
Fig S7, S15	mCit(S1h)4-gs	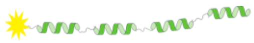	38.0

Fig S7	mCit(S1h)8-gs	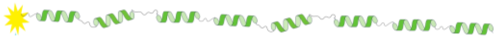	49.3
Fig S7	mCit(S1h)12-gs	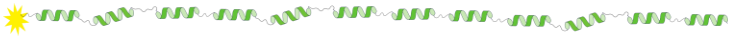	60.5
Fig S7	mCit(S1h)8-gs40	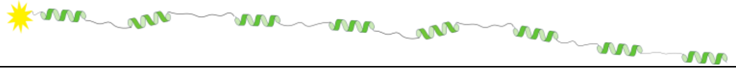	63.6
Fig S7, S15	TagBFP(S2h)4-gs	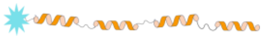	37.3
Fig S7	TagBFP(S2h)8-gs	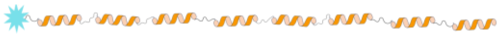	48.7
Fig S7	TagBFP(S2h)12-gs	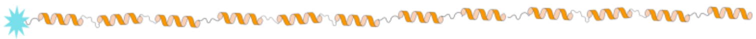	60.1
Fig S8	mCit(D1h-D3h)3-gs	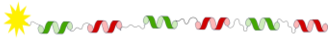	39.0
Fig S8	TagBFP(D2h)3(D4h)3-gs	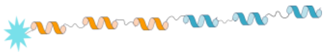	38.3
Fig 4, S9, S12	mCit-APPAP-gs	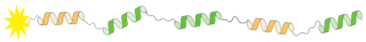	42.0
Fig 4, 5, S9, S12, S13	mCit-APPAPAPPAP-gs	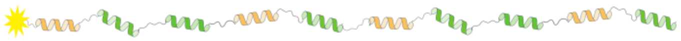	57.4
Fig 4, S12	mCit-PPPPPPAAAA-gs	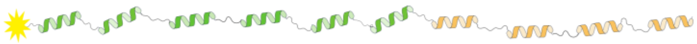	57.2
Fig S15	Cit(P5f)4-gs	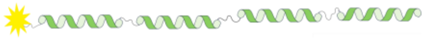	41.3
Fig S15	BFP(P6f)4-gs	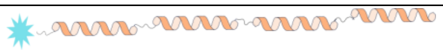	40.6

Supplementary Table 3: Helical propensity of coiled coils calculated with Agadir[1] algorithm.

**CC:**	**Helical content prediction according to Agadir algorithm:**
S1	2.11
S2	3.74
S3	2.04
S4	1.22
SAH	89.21
SAH-S1	70.66
SAH-S1-SAH	80.53
SAH-S3	61.89
SAH-S3-SAH	77.27
S1h	7.56
S2h	8.06
S3h	10.49
S4h	2.85

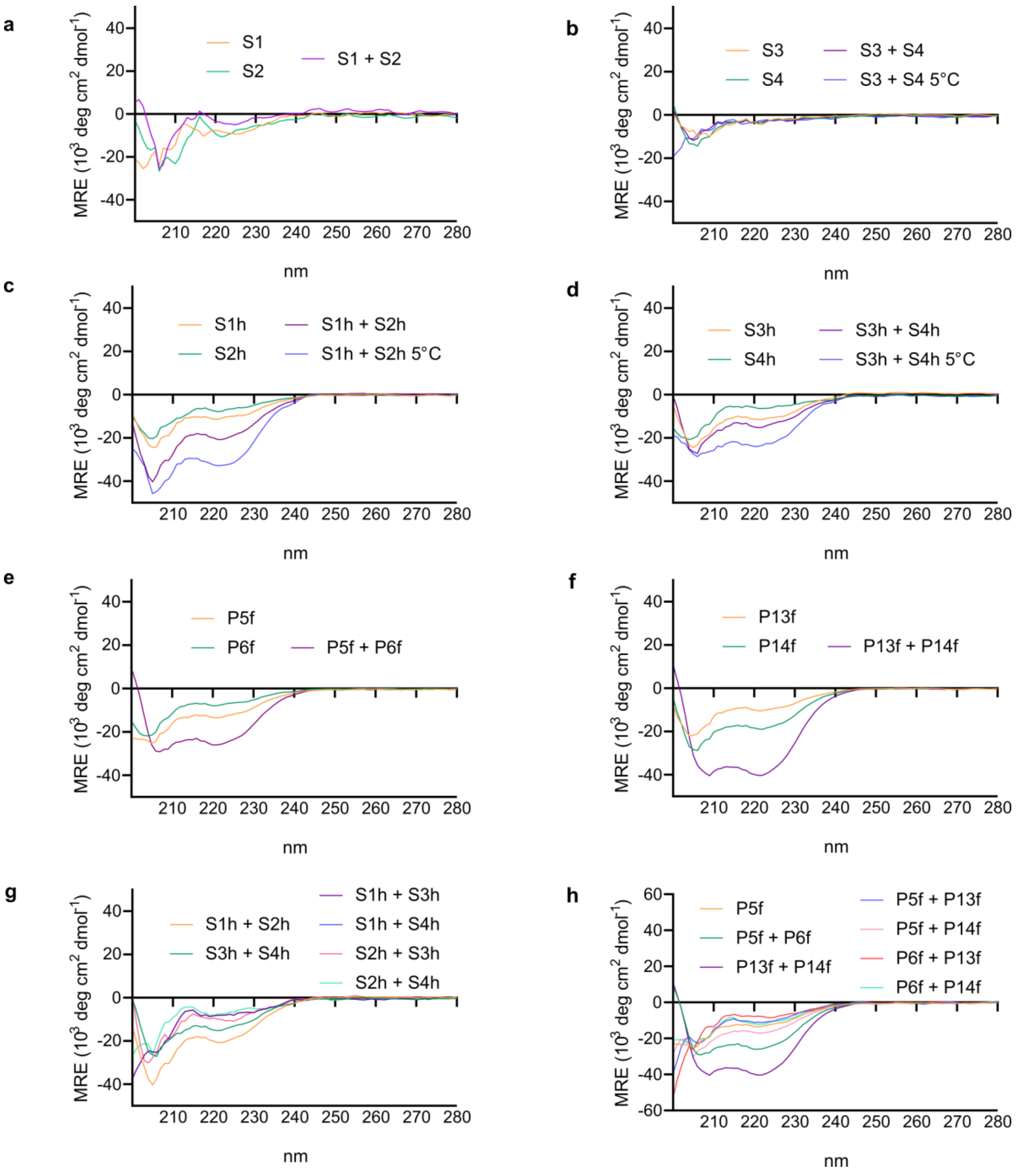

Supplementary Figure 1: Circular dichroism (CD) spectra of a 1:1 mixture of CC compared to spectra of a single peptide. a-f) Single peptides (40 µM) are shown in yellow and green. Peptide pairs (20 µM each) are shown in purple. All measurements were done at 20°C, except in b, c and d where a measurement at 5°C is included in blue. g, h) Spectra depicting orthogonality, showcasing the comparison between peptide pairs and non-pair peptide combinations for g) 3-heptad peptides and h) 4-heptad peptides measured at 20°C. The designated peptide partners exhibited a higher helical content than non-designated partners, indicating peptides' intrinsic preference for binding to their designated partners. All data were measured in Tris buffer. Source data are provided as a Source Data file.

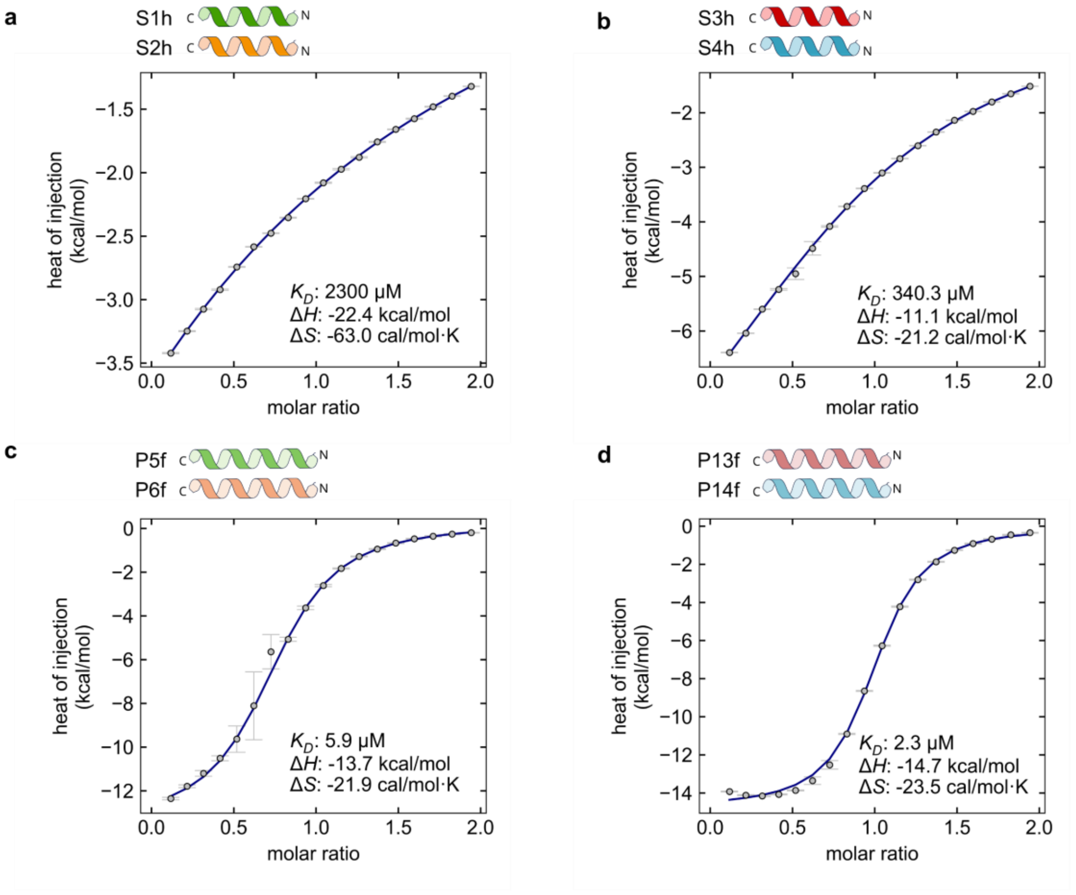

Supplementary Figure 2: Binding analysis between the weak 3-heptad and strong 4-heptad CC forming segment using isothermal titration calorimetry (ITC).

a) For peptide pair S1h + S2h peptide concentration in 40 µl injection syringe was 5.6 mM for S1h and concentration of complementary peptide S2h in ITC cell (volume 200 µl) was 560 µM. The Kd is 2.3 ± 1.5 mM and ΔH -22.4 ± 1.4 kcal/mol. b) For peptide pair S3h + S4h concentration in 40 µl injection syringe was 5.4 mM S3h and concentration of complementary peptide S4h in ITC cell (volume 200 µl) was 540 µM. The Kd is 0.34 ± 0.07 mM and ΔH -11.1 ± 0.5 kcal/mol. For peptide pair c) P5f + P6f and d) P13f + P14f, which are 4-heptad CC. Peptide concentration in 40 µl injection syringe was 1 mM for P5f and P13f and concentration of complementary peptide in the ITC cell was 100 µM P6f or P14f. c) The obtained Kd is 5.9 ± 0.5 µM and ΔH -13.7 +- 0.6 kcal/mol and d) Kd 2.3 ± 0.2 µM and ΔH -14.7 ± 0.6 kcal/mol. All the peptides were dissolved in a matching buffer 50 mM Tris pH of 7.5, 150 mM NaCl, and 1 mM TCEP.

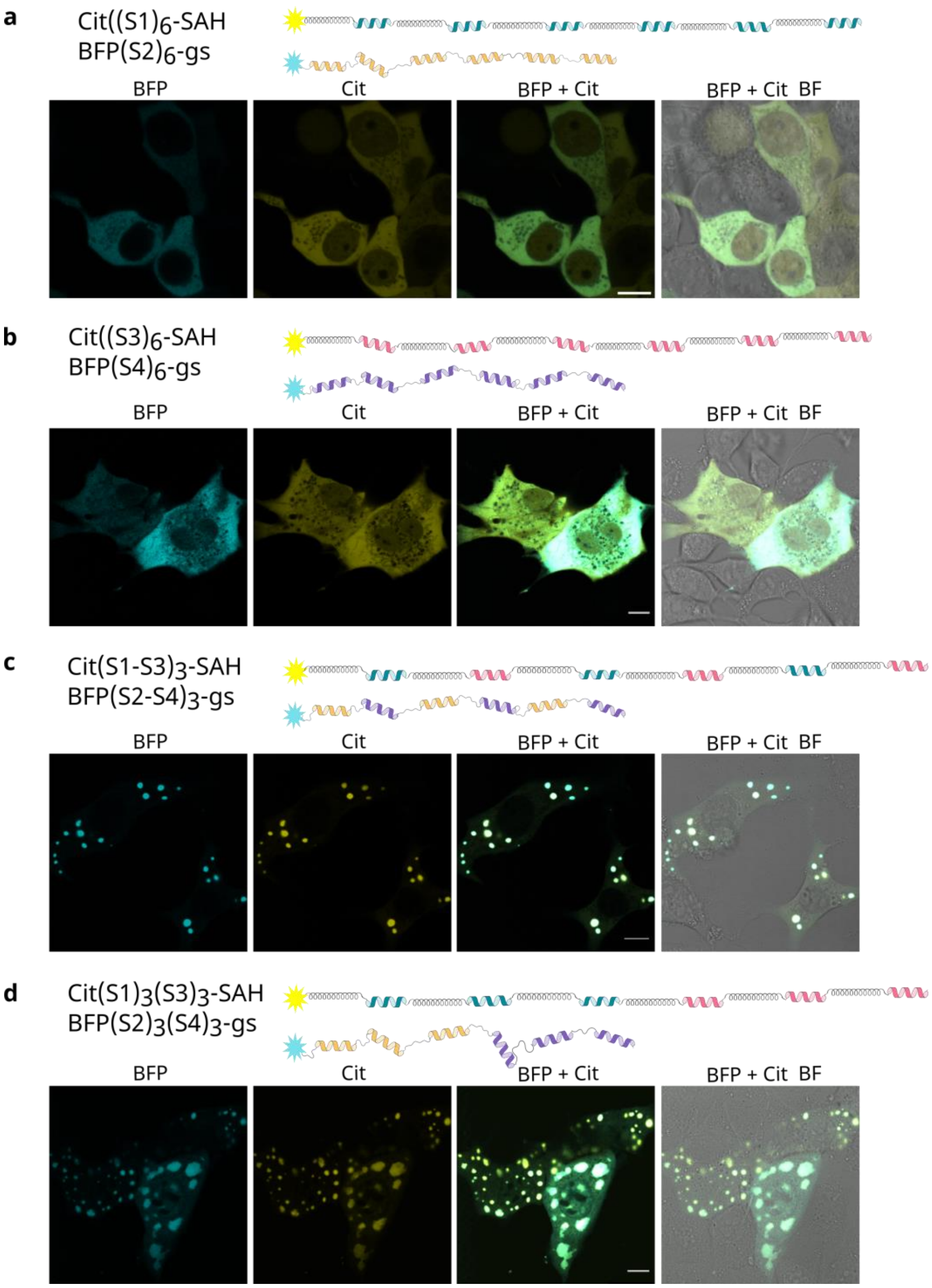

Supplementary Figure 3: Condensates are formed if both polypeptides have the same arrangement of CC, but not in case of a single CC pair type.

a) HEK293T cells expressing polypeptides with only a single type of CC. In the first mCitrine followed by six repeats of S1, separated with SAH linker. The second peptide TagBFP followed by six repeats of S4 separated with gs linker. b) NIH-3T3 cells expressing polypeptides with only a single type of CC. In the first mCitrine followed by six repeats of S2, separated with SAH linker. The second peptide TagBFP followed by six repeats of S4 separated

with gs linker. c) NIH-3T3 cells expressing polypeptides with mCitrine followed by S1 and S3 CC interchangeably repeated three times and separated with SAH linker. The second polypeptide is TagBFP followed by three repeats of S2 and S4 also interchangeably repeated, separated with gs linker. In the third picture both channels are merged, and the fourth picture is merge of both channels together with bright field. d) Same as c) only both polypeptides have segregated arrangement of CC (3+3). Scale bars, 10 μm.

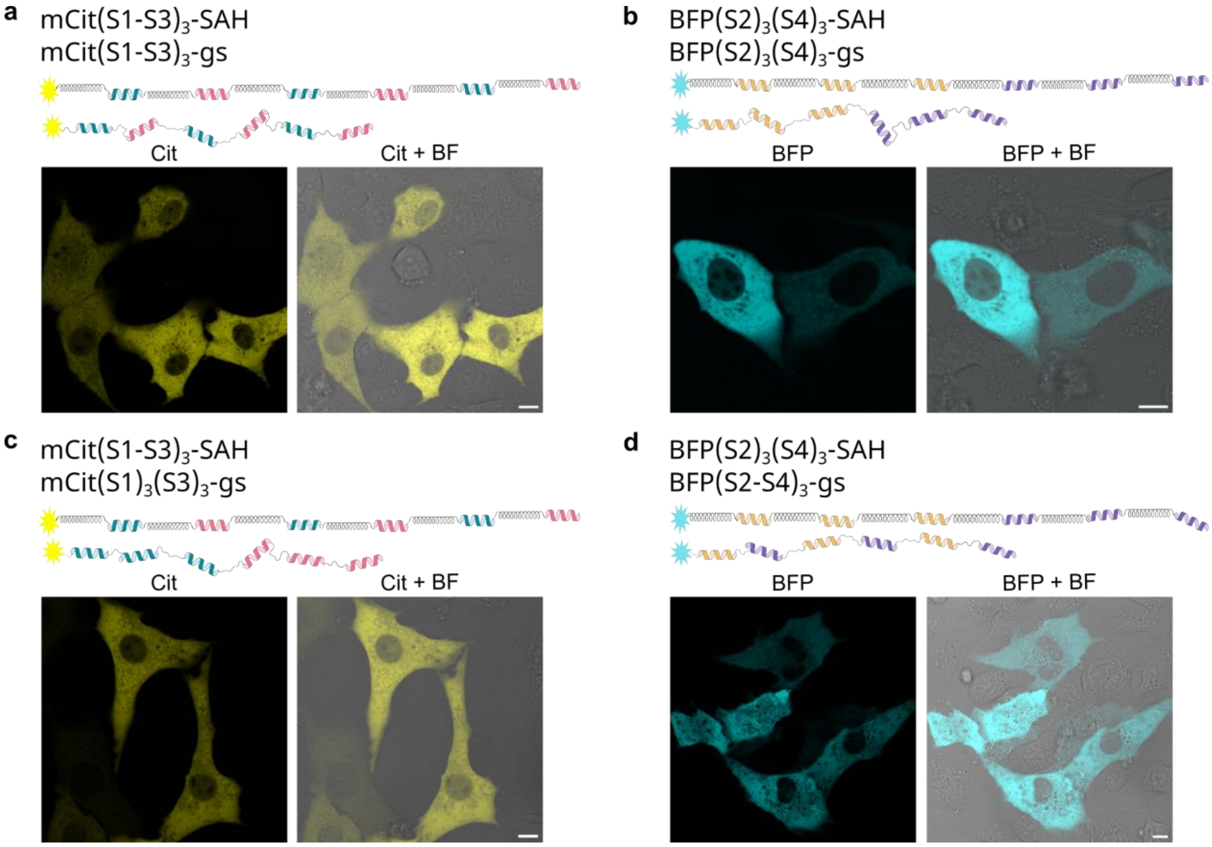

Supplementary Figure 4: Polypeptides containing the same CC type do not form condensates in cells.

a) mCitrine followed by repeats of S1 and S3 interchangeably repeated and separated with SAH linker and mCitrine followed by repeats of S1 and S3 interchangeably repeated and separated with gs linker. b) TagBFP followed by three S2 and three S4 separated with SAH linker and TagBFP followed by three S2 and three S4 separated with gs linker. c) NIH-3T3 cells expressing polypeptides both with mCitrine followed by repeats of S1 and S3 interchangeably repeated and separated with SAH linker and mCitrine followed by three repeats of S1 and three repeats of S3 separated with gs linker. d) TagBFP followed by three S2 and three S4 separated with SAH linker and TagBFP followed by three repeats of S2 and S4 interchangeably repeated and separated with gs linker. NIH-3T3 cells transfected with 100 ng of each plasmid. Scale bars, 10 μm.

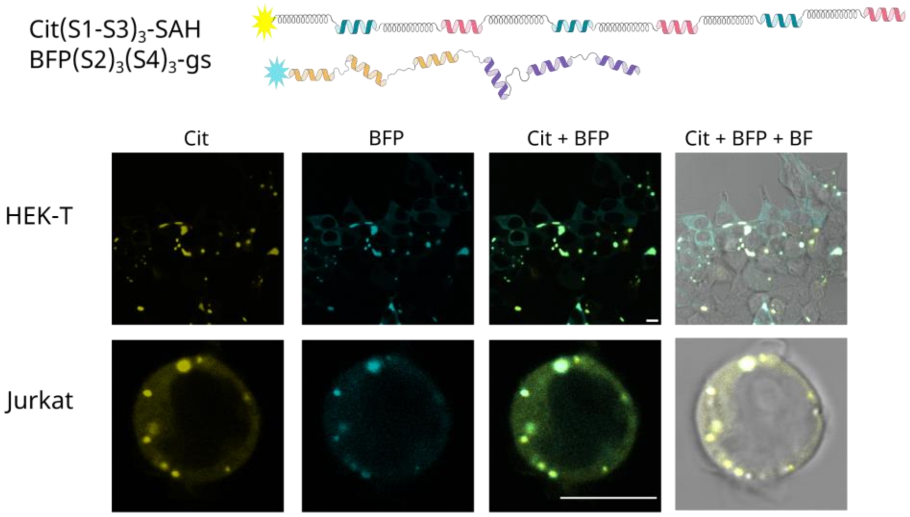

Supplementary Figure 5: Condensates form in various cell types.

HEK293T cells (above) and Jurkat cells (bellow) were transfected with a combination of plasmids (100 ng) each encoding one of the condensate-forming proteins. Scale bars, 10 µm.

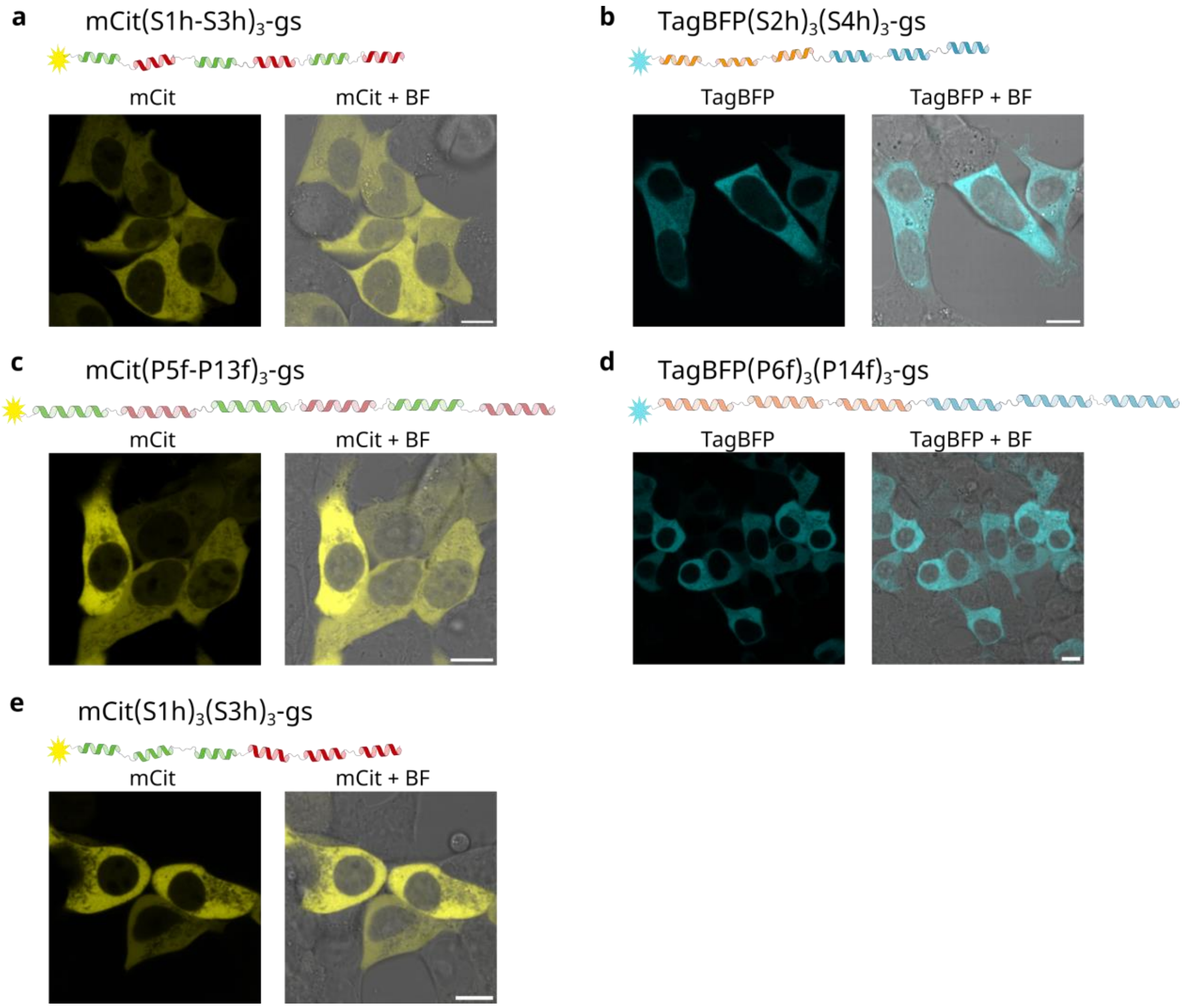

Supplementary Figure 6: Each polypeptide alone does not form condensates.

a-e) HEK293T cells transfected with 100 ng of plasmid encoding for single polypeptide. Scale bars, 10 μm.

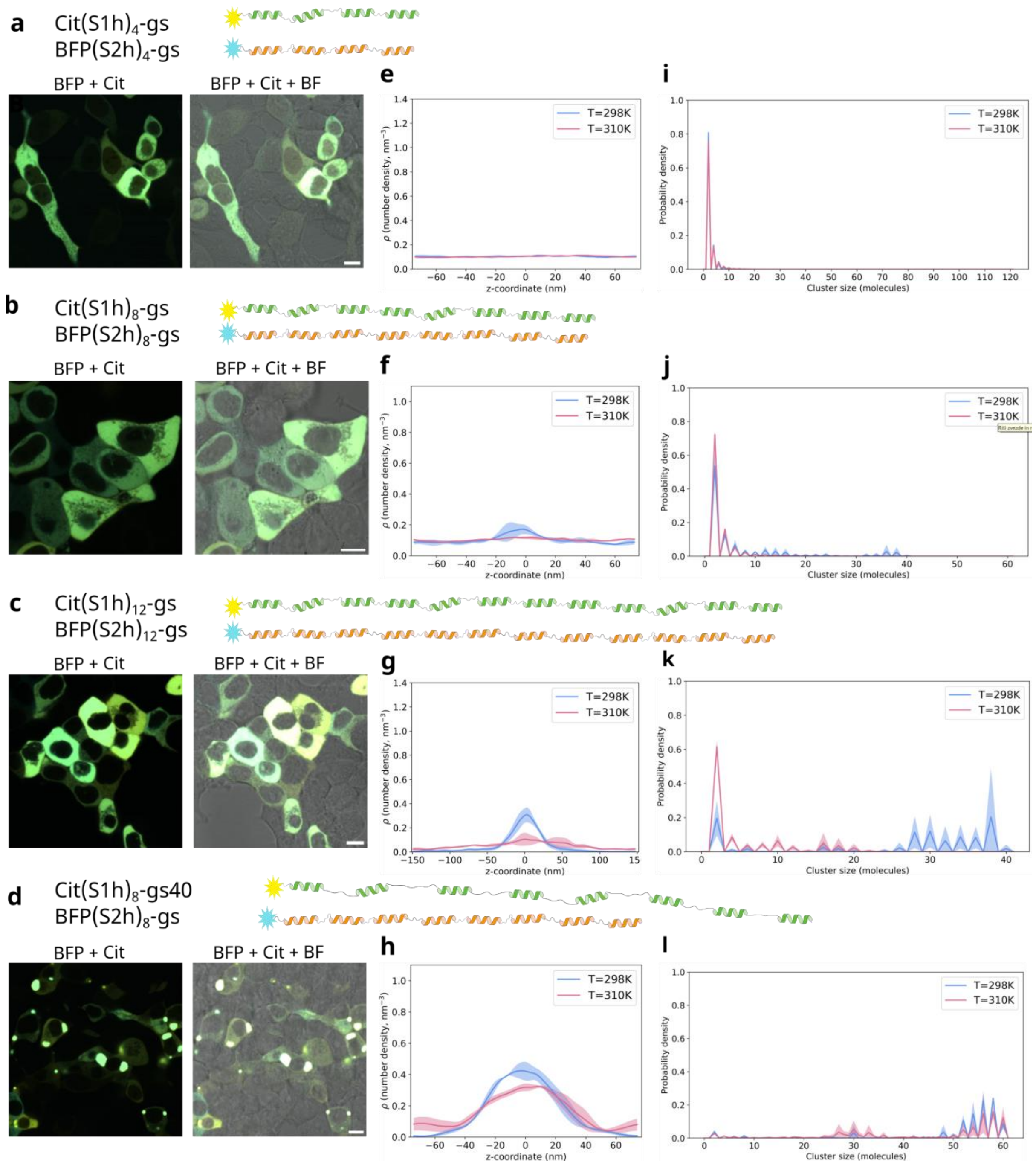

Supplementary Figure 7: Single CC type polypeptides with CC of higher helical propensity do not form condensates in cell or in simulation.

a-d) HEK293T cells transfected with 100 ng of each plasmid encoding polypeptide with a single CC type. The first protein Cit followed by 4 (a), 8 (b), 12 (c) repeats of S1h, separated by gs linker. In d) gs linker is 40 amino acids long. The second protein TagBFP followed by 4 (a), 8 (b), 12 (c) repeats of S2h, separated by gs linker. First picture both fluorescent channels are merged and the second picture both fluorescent channels together with bright field. Scale bars, 10 µm. e-h) Density profile analysis of simulated proteins with the same coil type. The LLPS droplet for the (S1h)$_{12}$-gs & (S2h)$_{12}$-gs protein combination (f) is different because it

required a larger simulation box size than the other two protein combinations shown, to accommodate the size of the larger proteins. Lines are the mean, and the shaded regions are standard deviation (*N*=3 simulation replicates). i-l) Cluster size distributions of simulated proteins with the same coil type. Lines are the mean, and the shaded regions are standard deviation (*N*=3 simulation replicates).

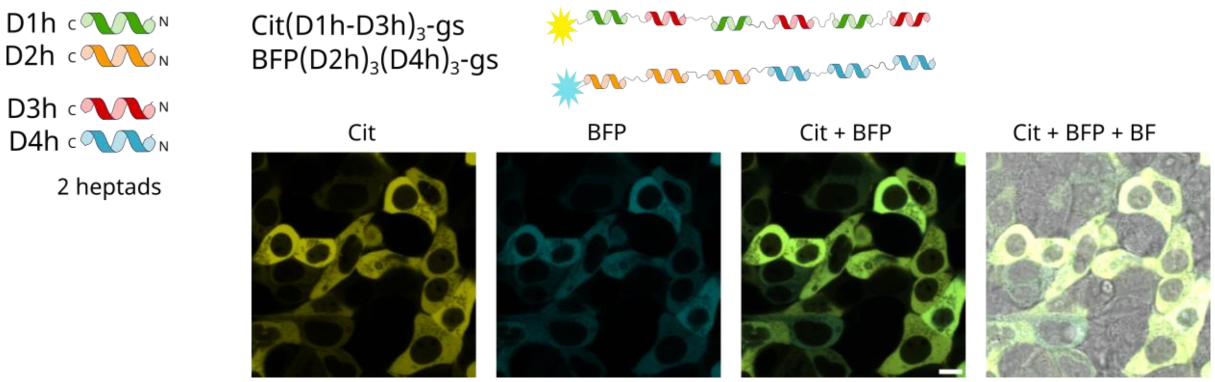

Supplementary Figure 8: Polypeptide pair with CCs that are 2 heptads long does not form condensates.

HEK293T cells transfected with 100 ng of each plasmid encoding polypeptides composed of 2 heptads long coiled coils with different arrangement and separated with gs linker. Scale bar, 10 µm.

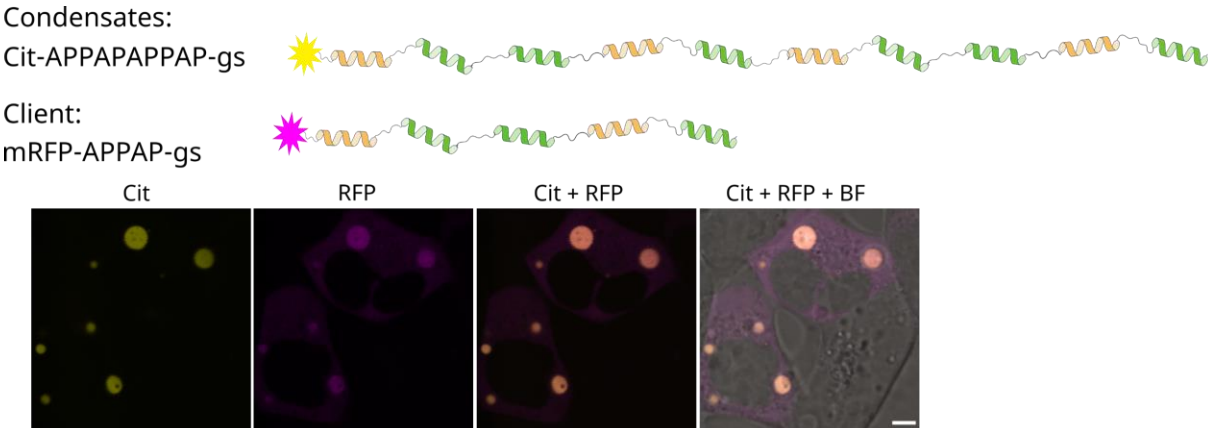

Supplementary Figure 9: Condensate formation incapable protein enriches into condensates and acts as client.

a) HEK293T cells expressing single chain polypeptide that forms condensates together with shorter polypeptide that is incapable of condensate formation. The first image is showing condensates labeled with mCit, second image shorter polypeptide labeled with mRFP that enriches to condensates. In the third both channels are merged and in the fourth both channels together with BF. Scale bar, 10 µm.

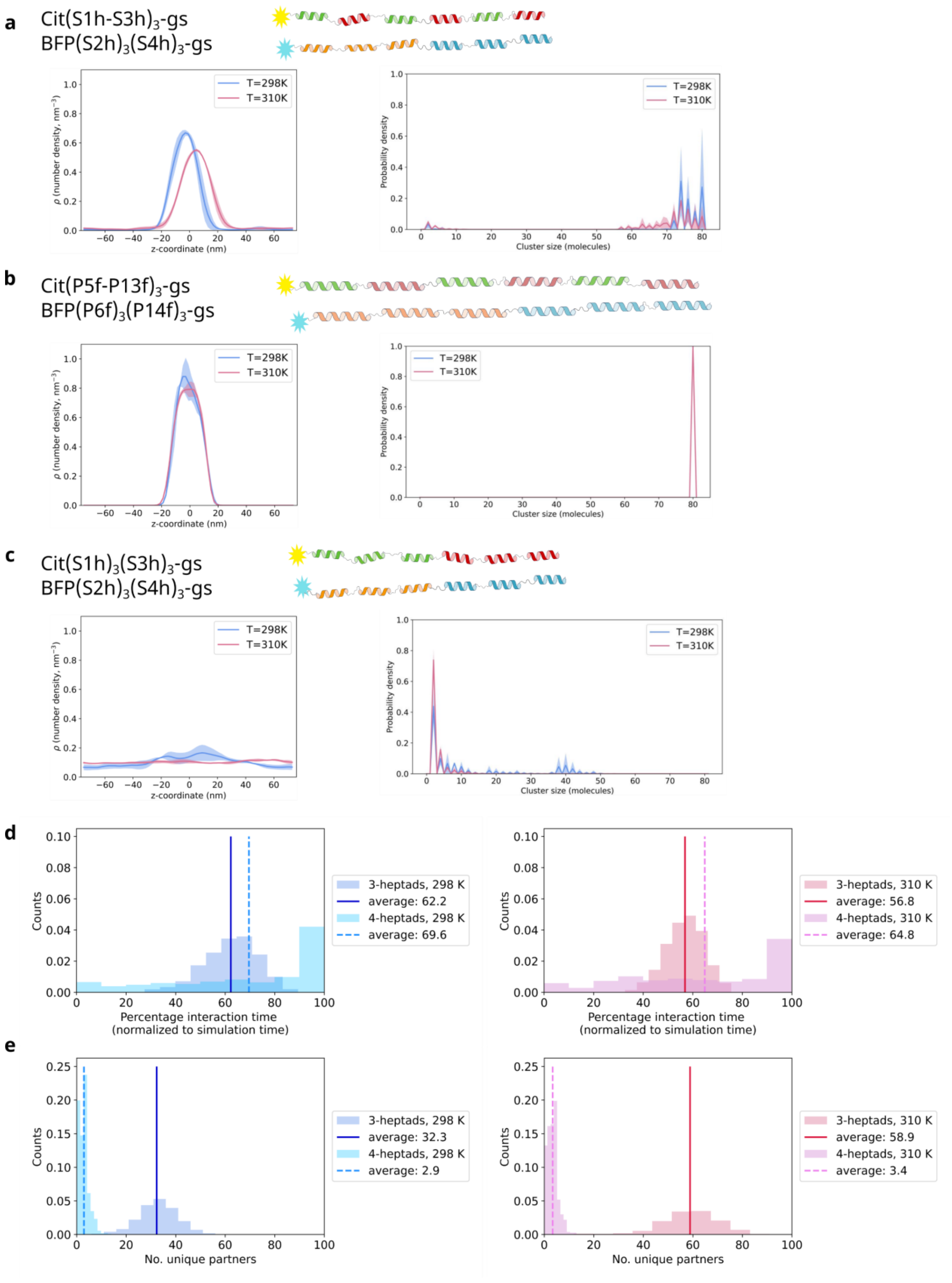

Supplementary Figure 10: Simulation LLPS propensity and droplet dynamics depend on the affinity of CC building modules and on their order in a polypeptide.

a) Density profile (left) and molecular cluster size distributions (right) of simulated proteins with 3-heptad long coils and different arrangement of CC between the polypeptides. b) Same

analyses in (a) but for proteins with 4-heptad long coils and different arrangement of CC between the polypeptides. c) Same analyses in (a) but for proteins with 3-heptad long coils and the same arrangement of CC between the polypeptides. Lines are the mean, and the shaded regions are standard deviation (*N*=3 simulation replicates) for plots in (a-c). d) Percentage of time that individual coil segments are engaged in a coil-coil interaction, left at 298 K and right at 310 K. Distributions are from compiled results from *N*=3 simulation replicates. Vertical lines represent the average across three replicates. Counts are normalized within each respective group. e) Number of unique partners that each coil segment interacts with throughout a simulation, left at 298 K and right at 310 K. Distributions are from compiled results from *N*=3 simulation replicates. Vertical lines represent the average across three replicates. Counts are normalized within each respective group.

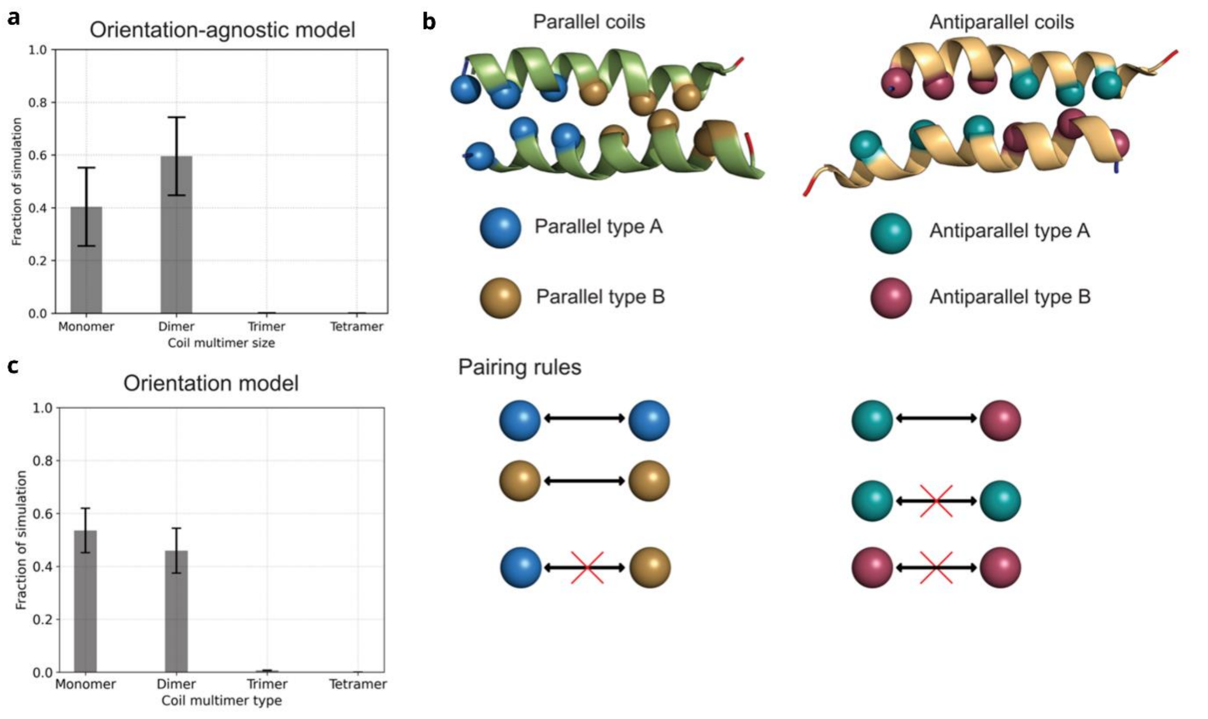

Supplementary Figure 11: Implementation of orientation-specific coils to the CC LLPS framework.

a) Quantification of multimer species in a small-scale simulation of $(S1h)_4$-gs and $(S2h)_4$-gs. Multimer counts are normalized to the total number of coils. Data are presented as mean, and error bars represent standard deviation from *N*=3 replicate simulations. b) Schematic illustrating the implementation of orientation-specific coil beads in to the CC LLPS framework. Colors of cartoon ribbons matches the coil specific coloring in Supplemental Table 1. The ends of each cartoon coil are colored deep blue, for the N-terminus, or bright red, for the C-terminus, to clarify the orientation of coils relative to each other. Colored beads along the cartoon ribbons show the orientation specific beads, along with the pairing rules used to enforce proper orientation. c) Quantification of multimer species in a small-scale simulation of orientation-specific mimic protein with coil arrangement PAPA. Multimer counts are normalized to the total number of coils.

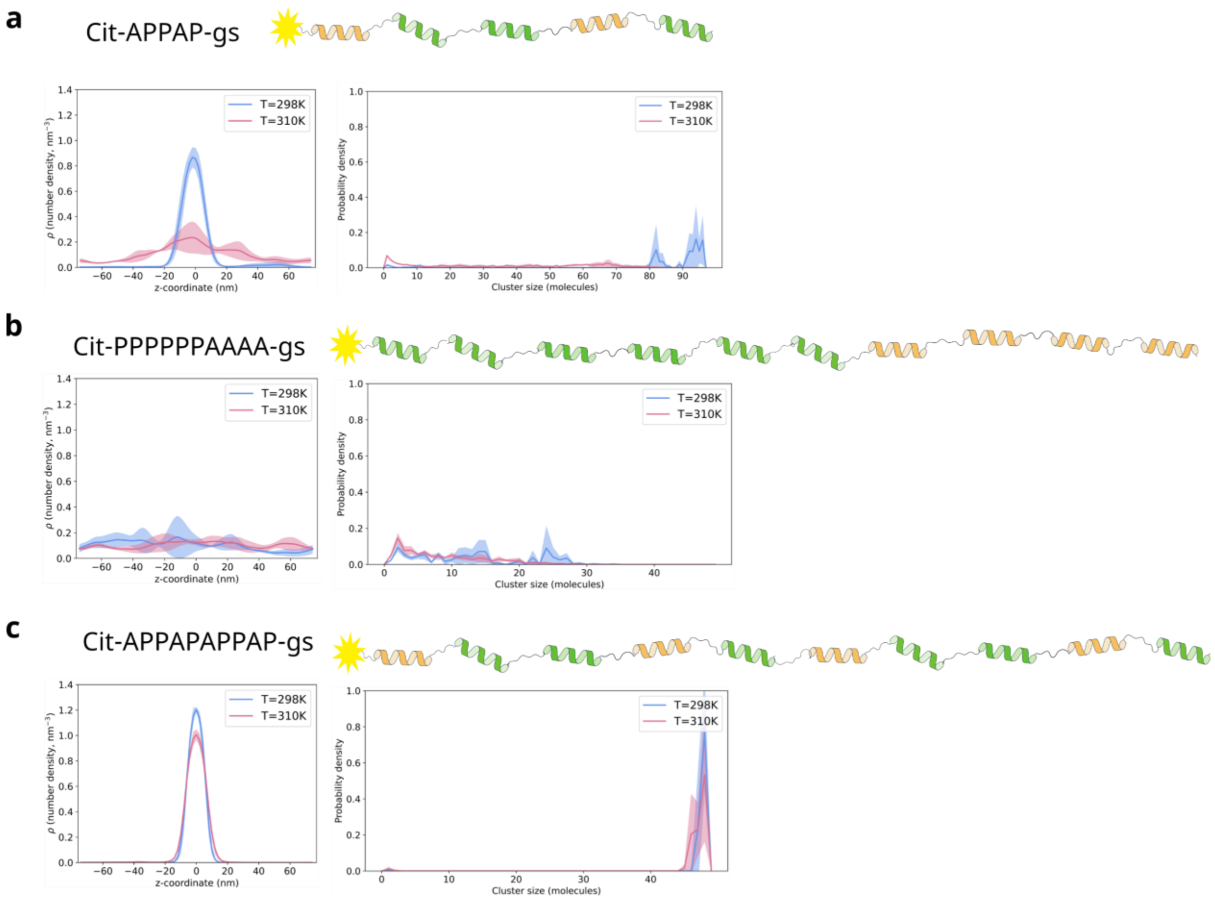

Supplementary Figure 12: Single chain polypeptides can form LLPS condensates *in silico*. Density profile (left) and molecular cluster size distributions (right) of a simulated protein with a) APPAP arrangement of coils b) PPPPPPAAAA arrangement of coils and c) APPAPAPPAP arrangement of coils. Lines are the mean, and the shaded regions are standard deviation (N=3 simulation replicates).

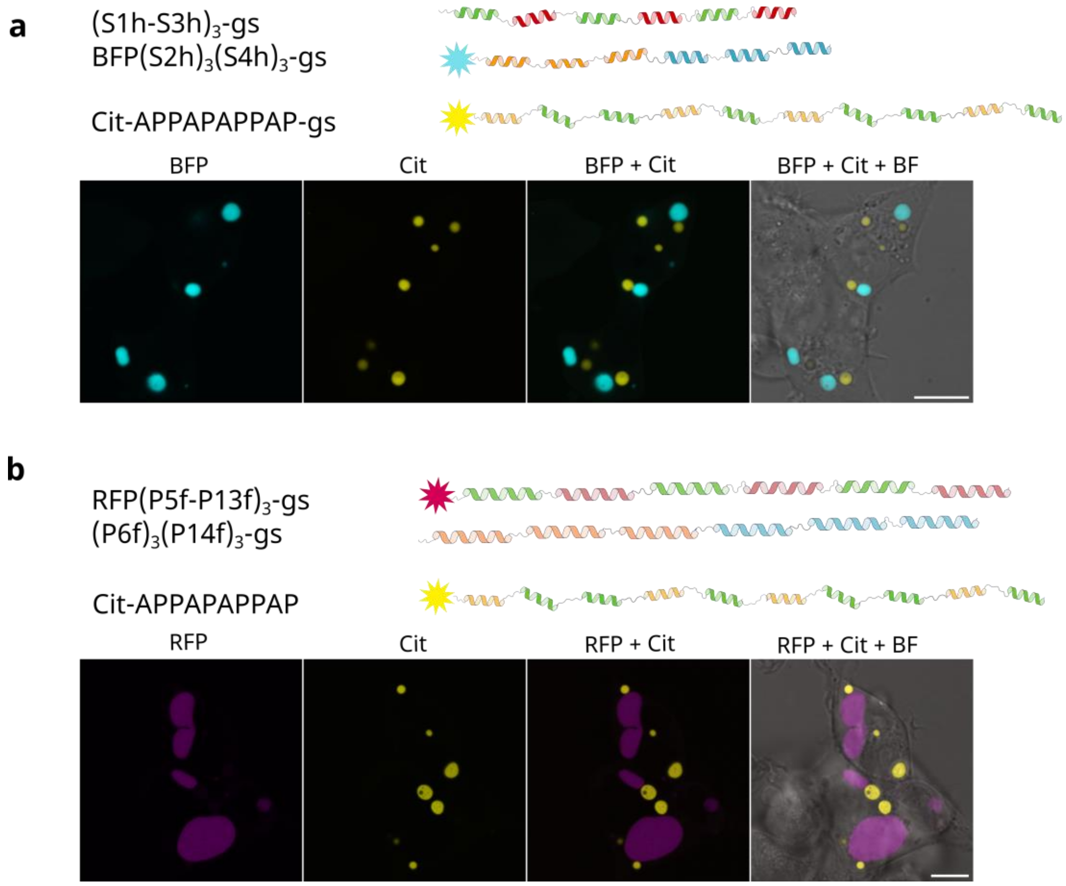

Supplementary Figure 13: More orthologous condensate can exist in a single mammalian cell.

a) HEK293T cells transfected with 100 ng of each plasmid encoding polypeptides. First polypeptide pair forming condensates with differential arrangement of 3 heptad CC, separated with gs linker. First polypeptide of the pair is without fluorescent protein, so the formed condensates are cyan. Second are single polypeptide chain condensates in labeled with mCitrine. b) HEK293T cells transfected with 100 ng of each plasmid encoding polypeptides. First polypeptide pair forming condensates with differential arrangement of 4 heptad CC, separated with gs linker. One polypeptide of the pair is without fluorescent protein, the other is with TagRFP (magenta). Second are single polypeptide chain condensates in labeled with mCitrine. Scale bars, 10 μm.

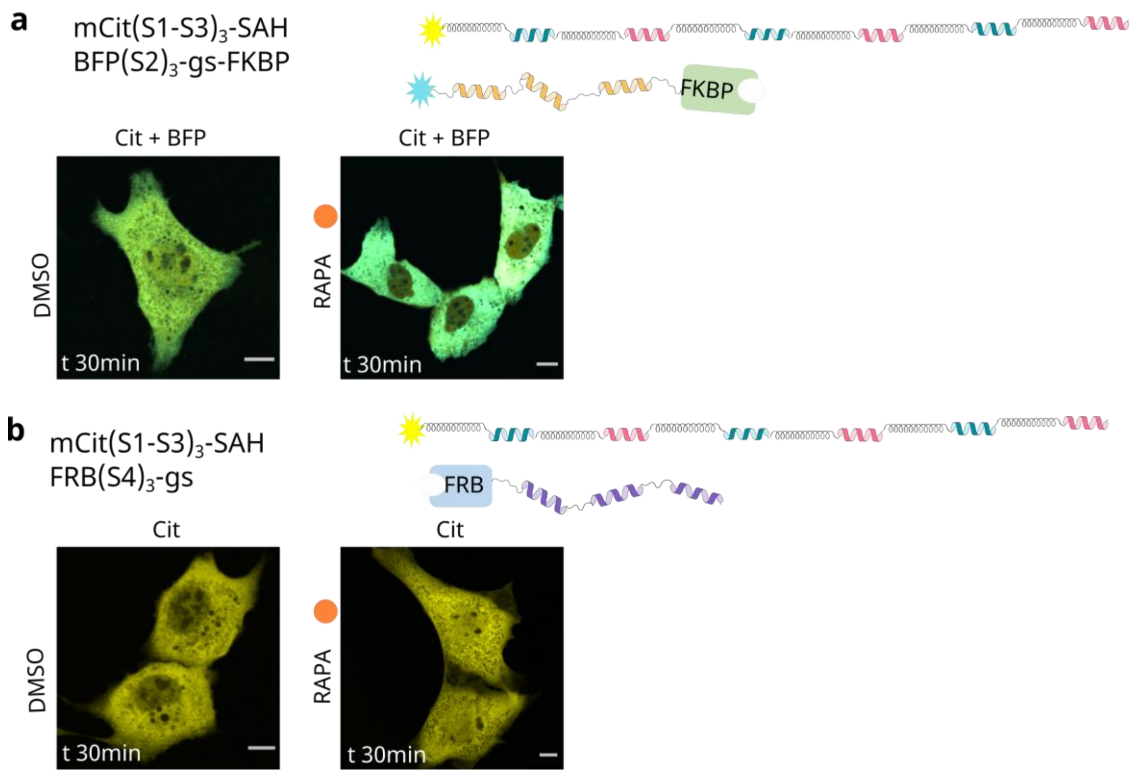

Supplementary Figure 14: No condensates form when cells are transfected with only one part of the second polypeptide chain.

a) NIH-3T3 cells transfected with 100 ng of each plasmid, first encoding for the whole polypeptide from a condensate forming pair and the second one part of the second polypeptide with FKBP heterodimerization domain. First image is taken 30 min after addition of DMSO and the second image 30 minutes after addition of rapamycin. b) NIH-3T3 cells transfected with 100 ng of each plasmid, first encoding for the whole polypeptide from a condensate forming pair and the second one part of the second polypeptide with FRB heterodimerization domain. First image is taken 30 min after addition of DMSO and the second image 30 minutes after addition of rapamycin. Scale bars, 10 μm.

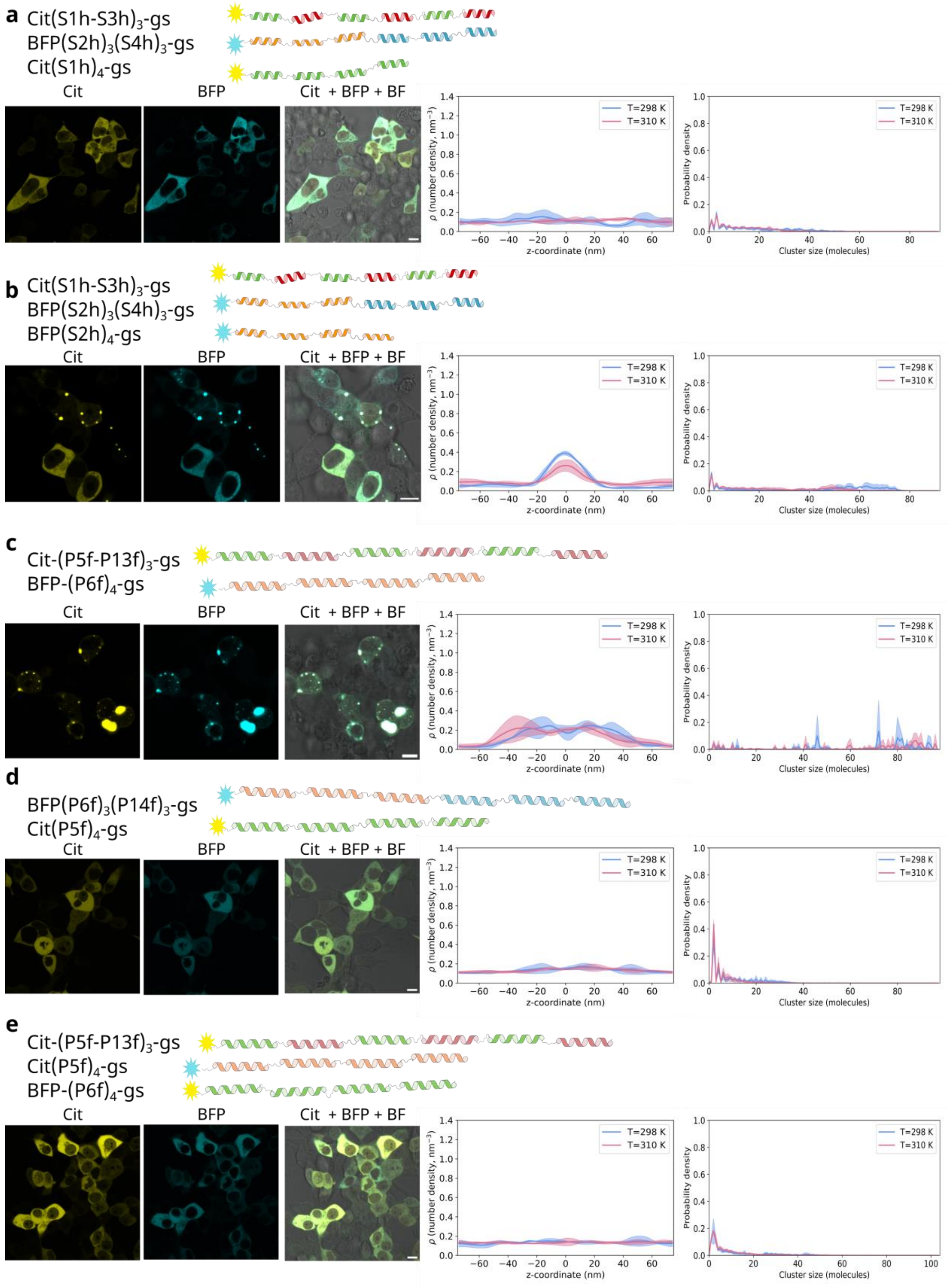

Supplementary Figure 15: CC-LLPS condensate formation can be regulated by intentional formation of molecular dimers.

In all five subfigures (a-e), a schematic of the tested polypeptides along with their names are provided. Representative microscopy images shown as three image panels, with the mCit only channel (left-most), BFP only channel (middle), and the mCit-BFP merged channel (right-most). Scale bars are 10 μm. Results from predictive simulations are shown as density profile plots (left-most plot, with high density and low density regions indicating LLPS) and molecular cluster distributions (right-most plot), with high-aggregate numbers (40-100) indicating LLPS. In all cases, predictive simulations and experiments qualitatively agree on whether or not LLPS occurs. Solid lines represent the mean and shaded regions are the standard deviation from N=3 replicate simulations.

Supplemental Methods

CC LLPS framework details specific to this study

Protein interactions occur between coil segments that belong to the same pair – e.g. S1h interacts with S2h but not S3h etc. – to preserve the same type of interactions observed *in vitro*. There are no attractive interactions outside of those between coil segments. We use the same interaction terms in our simulations as in the original CC LLPS framework, with the exception that sticky interactions between coil segments use $\sigma = 0.570$, and $\varepsilon = 7.5$ kJ/mol. We found these interaction parameters resulted in coil segments specific to this study to form dimers at similar strength to the original CC LLPS framework (Supplementary Figure 12A). We ran small scale simulations of the $(gs\text{-}S1h)_4$ and $(gs\text{-}S2h)_4$ protein pair to validate the listed interaction parameters. We ran single molecule MD simulations of individual proteins for 5 μs at 298 K, similar to the protocol for preparing structures for the slab method (see below). We packed 4 copies each of $(gs\text{-}S1h)_4$ and $(gs\text{-}S2h)_4$ using the configuration from the last frame of the single molecule MD into a 25 x 25 x 25 nm box. We ran triplicate simulations starting from the packed box, which included equilibrating the boxes at 298.15 K for 100 ns in the NVT ensemble, then running production simulations at 298.15 K in the NVT ensemble for 2 μs. We counted the population of each multimer over every frame for each replicate starting at 1 μs, then averaged the multimer populations across the three replicates.

We also generated an orientation-specific version of the framework to allow for control over parallel versus antiparallel CC interactions. We altered the coil sticky beads in the original CC LLPS framework to make orientation specific sticky beads such that parallel coils will only interact with parallel coils, and antiparallel will only interact with antiparallel. The new orientation-specific beads also enforce proper orientation. A schematic demonstrating how the orientation-specific beads are implemented in each type of coil, as well as a diagram of how orientation-specific coils interact, is shown in Supplementary Figure 12B**.** We use $\sigma = 0.570$, and $\varepsilon = 8.0$ kJ/mol and the interaction parameters for the orientation-specific coil sticky beads. These parameters result in coil interactions that most closely resemble the interactions seen for the regular coil sticky beads described above (Supplementary Figure 12A, 12C). We optimized these parameters using small scale simulations of protein models that mimic the single polypeptide constructs used *in vivo*. We constructed a peptide with 4 coils (each with 3 heptads), connected with gs-linkers, with the following orientation topology: P-AP-P-AP, where "P" is a parallel coil, and "AP" is antiparallel. We generated a coarse-grained structure using the PeptideBuilder strategy[2]. We then packed eight copies of this peptide into a box sized

25 x 25 x 25 nm. We did not run single molecule simulations prior to packing. We ran triplicate simulations with varying interaction parameters, starting from the packed box. We equilibrated the box for 100 ns in NVT ensemble at 298.15 K, then ran production simulations for 2 μs in the NVT ensemble at 298.15 K. We quantified the population of each multimer over every frame for each replicate starting at 1 μs, then averaged the multimer populations across the three replicates (Supplementary Figure 12C).

Slab protocol for phase coexistence

Single molecule simulations to generate configurations for slab simulations:

We used the same method in Ramirez et al. [2023] to generate the initial 3D representation of our desired proteins. Generated proteins were then placed in a simulation box large enough to accommodate its initial size. We performed single molecule MD of each protein to equilibrate its structure and to generate a library of configurations. Each protein was energy minimized, equilibrated in the NVT ensemble for 500 ps with a time step of 25 fs, then production simulations were done for 5 μs in the NVT ensemble with a time step of 25 fs. Single molecule MD simulations were performed at each of the temperatures used in this study. Only 1 replicate single molecule simulation was performed.

Preparing starting simulation box for slab simulations:

Starting slab simulation boxes were generated by packing copies of each desired protein using configurations generated from the single molecule MD. All slab simulations had a constant 480 coil segments per box. This standardized the simulations and allowed us to make comparisons between different protein pairs. We randomly selected 5 separate configurations for each protein from the equilibrated single molecule MD (equilibrated trajectories start at 100 ns for all proteins). We used Random.org to randomly select the frames and corresponding configurations. Each of the 5 configurations were copied so that each simulation contained the desired final coil segment density (480 per box). For simulations of protein pairs, each protein is present in 1:1 stoichiometry. Packmol version 20.11.1[3] was used for packing, with a tolerance of 1.0 nm between each protein copy, in a box sized 30x30x200 nm. We generated three unique packed boxes (with unique configurations) to generate three replicate slab simulations for all protein combinations investigated, at each temperature (298 and 310 K).

Slab simulations:

Packed boxes were energy minimized, and then equilibrated in the NPT ensemble for 200 ns with a time step of 20 fs at 150 K. Semi-isotropic pressure coupling was applied only in the z-dimension of the box using the Parinello-Rahman barostat, with a reference pressure of 1 bar,

compressibility of $3*10^{-4}$ bar^{-1}, and a time constant of 5 ps for pressure coupling. At the end of NPT equilibration, the boxes were compressed to approximately 20% of the starting z-axis length, and at this point the slab is formed. Proteins crossing periodic boundaries are made whole then the z-axis is expanded to a total length of 150 nm. We found that proteins with 12 coil segments per chain were too big to observe LLPS in a 150 nm long box, and so a 300 nm long box was used for these proteins. The x- and y-dimensions remained fixed at 30 nm long for all slab simulations. Slabs were then equilibrated in the NVT ensemble for 200 ns with a time step of 20 fs at the desired temperature. We performed production MD simulations for each temperature-equilibrated slab for 20 μs with a time step of 25 fs. Proteins with 12 coils per chain, 40-bead long linkers, or 4 heptad long coil segments were simulated for a total of 40 μs per slab per replicate. Equilibrium was assessed by monitoring the density of both the center of the box, and the starting and ending edges of the box combined. Equilibrium occurs when both these regions have unchanging density. Only the equilibrated portions of trajectories were used for analysis. Each slab simulation had at least 10 μs of equilibrated trajectory for analysis.

Density profile analyses

We performed density analysis of each equilibrated trajectory using the `gmx_density` module in GROMACS. Density (reported as number density / nm^3) was calculated in 2 nm slices along the z-axis of simulations with 150 nm long boxes, and in 4 nm slices along the z-axis of simulations with 300 nm long boxes. The average density in each slice for a trajectory is output. Density profiles along the z-axis for all protein pairs, at a given temperature, were averaged across three replicate simulations and are plotted as mean (solid lines) +/- standard deviation (shaded regions). These data are used in determining LLPS in combination with molecular cluster analysis.

Molecular cluster analyses

Molecular cluster size distributions were quantified from every 10 ns of equilibrated trajectories using the `gmx_clustsize` module in GROMACS. Cluster sizes for whole molecules were calculated using a distance cut-off of 0.9 nm. The output cluster size distributions were normalized to probabilities. Cluster size probability distributions were averaged across three replicate simulations, for each protein pair at each temperature, and are reported as mean (solid lines) +/- standard deviations (shaded regions). Molecular clusters containing nearly all of the proteins in a simulation, in combination with density transition (revealed by density profile analysis), is the signature of LLPS.

Coil interaction analyses

We used the same multimer-counting strategy in[2] to analyze the multimer populations in our simulations. Our approach calculates the distances between each coil segment's center of mass and classifies multimers by a distance cutoff of 1.3 nm. We use this method to validate the interaction parameters used in this study (described above), as well as to do interaction time and unique partners analysis for individual coil segments. The custom analysis scripts to do these analyses are located on the GitHub repository associated with this paper.

Mean square displacement and diffusion coefficient analyses

Mean square displacement (MSD) of individual protein copies in equilibrated simulations were calculated using the `gmx_msd` module in GROMACS. MSD was calculated from the center of mass of each protein, and calculated every 10 ns from equilibrated trajectories. Molecules were made whole, and periodic boundary conditions were taken into account for the calculation. The starting frame of the equilibrated trajectory was used as the reference frame. The MSD of each individual protein was averaged to produce a system MSD from which the diffusion coefficient was calculated. Three replicate system MSD data were generated for each protein pair analyzed at both temperatures. We performed bootstrapping to estimate the diffusion coefficient and the standard deviation. 450 data points were sampled with replacement randomly from any of the three replicate MSD data sets. Only MSD data from 0.51 to 5.01 (μs) lag-times were used in the bootstrap, which corresponds from 5% to 50% of the MSD data. A line was fit to the set of sampled data and the diffusion coefficient was calculated from the estimated slope by dividing by six. The bootstrap was repeated 5000 times, and the average diffusion coefficient and standard deviation were calculated.

REFERENCES:

1. Lacroix, E., Viguera, A. R. & Serrano, L. Elucidating the folding problem of α-helices: local motifs, long-range electrostatics, ionic-strength dependence and prediction of NMR parameters 1 1Edited by A. R. Fersht. *J Mol Biol* **284**, 173–191 (1998).

2. Ramirez, D. A., Hough, L. E. & Shirts, M. R. Coiled-coil domains are sufficient to drive liquid-liquid phase separation of proteins in molecular models. *bioRxiv* (2023) doi:10.1101/2023.05.31.543124.

3. Martínez, L., Andrade, R., Birgin, E. G. & Martínez, J. M. PACKMOL: a package for building initial configurations for molecular dynamics simulations. *J Comput Chem* **30**, 2157–64 (2009).
