# Supplementary material for: Programmable de novo designed coiled coil-mediated phase separation in mammalian cells
Source: Nat Commun. 2023 Dec 2;14:7973. doi: 10.1038/s41467-023-43742-w (PMC10693550; doi:10.1038/s41467-023-43742-w)
Supplement: Supplementary file 3 — Description of Additional Supplementary Files [file 41467_2023_43742_MOESM3_ESM.pdf]

Title: Supplementary Movie 1:
Description: Liquid behaviour of condensates from Cit(S1-S3)3-SAH and BFP(S2)3(S4)3-gs transfected to NIH-3T3 cells, both 100 ng. Video is presenting merged yellow and cyan channel, picture taken sequentially every 15 seconds. Scale bar, 10 µm.

Title: Supplementary Movie 2:
Description: Liquid behaviour of condensates from Cit(S1h-S3)3-gs and BFP(S2h)3(S4h)3-gs transfected to HEK293T cells, both 100 ng. Video is presenting merged yellow and cyan channel, picture taken sequentially every 10 seconds. Scale bar, 10 µm.

Title: Supplementary Movie 3:
Description: Liquid behaviour of single polypeptide chain condensates Cit-APPAPAPPAP-gs transfected to HEK293T cells, 100 ng. Video is presenting yellow channel, picture taken every 10 seconds. Scale bar, 10 µm.

Title: Supplementary Movie 4:
Description: Inducible formation of condensates. NIH-3T3 cells transfected with the first of the pair (whole protein) Cit(S1-S3)3-SAH together with both parts of the second protein BFP(S2)3-gsFKBP and FRB(S4)3-gs. Time series after addition of 1 µM rapamycin. Video is presenting merged yellow and cyan channel, picture taken sequentially every 30 seconds. Scale bar, 10 µm.

Title: Supplementary Movie 5:
Description: Inducible formation of single polypeptide chain condensates. HEK293T cells transfected with both parts of the protein coding plasmids Cit-APPAP-gs-FKBP and FRB-APPAP-gs. Time series after addition of 1 µM rapamycin. Video is presenting yellow channel, picture taken every 20 seconds. Scale bar, 10 µm

Title: Supplementary Data 1:
Description: Amino-acid sequences of protein domains and peptides, used in the study.

Title: Supplementary Data 2:
Description: Oligonucleotide sequences.
