# Supplementary material for: Programmable de novo designed coiled coil-mediated phase separation in mammalian cells
Source: Nat Commun. 2023 Dec 2;14:7973. doi: 10.1038/s41467-023-43742-w (PMC10693550; doi:10.1038/s41467-023-43742-w)
Supplement: Supplementary file 11 — Reporting Summary [file 41467_2023_43742_MOESM11_ESM.pdf]

nature portfolio

Corresponding author(s): Roman Jerala
Last updated by author(s): Nov 12, 2023

Reporting Summary

Nature Portfolio wishes to improve the reproducibility of the work that we publish. This form provides structure for consistency and transparency in reporting. For further information on Nature Portfolio policies, see our Editorial Policies and the Editorial Policy Checklist.

Statistics

For all statistical analyses, confirm that the following items are present in the figure legend, table legend, main text, or Methods section.

n/a	Confirmed	
☐	☒	The exact sample size (*n*) for each experimental group/condition, given as a discrete number and unit of measurement
☐	☒	A statement on whether measurements were taken from distinct samples or whether the same sample was measured repeatedly
☒	☐	The statistical test(s) used AND whether they are one- or two-sided<br>*Only common tests should be described solely by name; describe more complex techniques in the Methods section.*
☒	☐	A description of all covariates tested
☒	☐	A description of any assumptions or corrections, such as tests of normality and adjustment for multiple comparisons
☐	☒	A full description of the statistical parameters including central tendency (e.g. means) or other basic estimates (e.g. regression coefficient) AND variation (e.g. standard deviation) or associated estimates of uncertainty (e.g. confidence intervals)
☒	☐	For null hypothesis testing, the test statistic (e.g. *F*, *t*, *r*) with confidence intervals, effect sizes, degrees of freedom and *P* value noted<br>*Give P values as exact values whenever suitable.*
☒	☐	For Bayesian analysis, information on the choice of priors and Markov chain Monte Carlo settings
☒	☐	For hierarchical and complex designs, identification of the appropriate level for tests and full reporting of outcomes
☒	☐	Estimates of effect sizes (e.g. Cohen's *d*, Pearson's *r*), indicating how they were calculated

Our web collection on statistics for biologists contains articles on many of the points above.

Software and code

Policy information about availability of computer code

Data collection	Leica TCS SP5 inverted laser-scanning microscope on a Leica DMI 6000 CS module, numerical aperture (NA) 1.4 (Leica Microsystems) was used to obtain micrographs and preform FRAP experiments. , Leica LAS AF software was used for image acquisition. Chirascan CD spectrometer (Applied Photophysics) was used for CD experiments. MicroCal PEAQ-ITC (Malvern) was used for isothermal titration calorimetry. Molecular dynamics simulations were performed using unmodified GROMACS v2022.1.
Data analysis	Custom analysis code was written in Python 3. 9: https://github.com/dora1300/programmable_coiledcoil_llps<br>Raw thermograms were integrated with the software NITPIC 2.1.0, interaction analysis was done with SEDPHAT, and titration curves were visualized with a companion GUSSI software.<br>Graphs and statistical analyses were prepared with GraphPad Prism.8.4.3<br>(http://www.graphpad.com/). The microscopy pictures were analyzed and prepared with LAS X 1.4.5, Leica (https://www.leica-microsystems.com/products/microscope-software/p/leica-las-x-ls/) and ImageJ 1.53t (https://imagej.nih.gov/ij/). Schemes were prepared with Inkscape™ 0.92.3, Brooklyn, NY (https://inkscape.org/).

For manuscripts utilizing custom algorithms or software that are central to the research but not yet described in published literature, software must be made available to editors and reviewers. We strongly encourage code deposition in a community repository (e.g. GitHub). See the Nature Portfolio guidelines for submitting code & software for further information.

Data

Policy information about availability of data

All manuscripts must include a data availability statement. This statement should provide the following information, where applicable:

- Accession codes, unique identifiers, or web links for publicly available datasets
- A description of any restrictions on data availability
- For clinical datasets or third party data, please ensure that the statement adheres to our policy

The data generated in this study and supporting the findings of this study are available in the paper and its Supplementary Information files. Source data are provided with this paper. Any other relevant data or reagents are available from the corresponding author upon reasonable request.

Research involving human participants, their data, or biological material

Policy information about studies with human participants or human data. See also policy information about sex, gender (identity/presentation), and sexual orientation and race, ethnicity and racism.

Reporting on sex and gender	N/A
Reporting on race, ethnicity, or other socially relevant groupings	N/A
Population characteristics	N/A
Recruitment	N/A
Ethics oversight	N/A

Note that full information on the approval of the study protocol must also be provided in the manuscript.

Field-specific reporting

Please select the one below that is the best fit for your research. If you are not sure, read the appropriate sections before making your selection.

☒ Life sciences ☐ Behavioural & social sciences ☐ Ecological, evolutionary & environmental sciences

For a reference copy of the document with all sections, see nature.com/documents/nr-reporting-summary-flat.pdf

Life sciences study design

All studies must disclose on these points even when the disclosure is negative.

Sample size	We chose sufficient sample size to establish that the values compared were derived from normal distributions. The number of replicates are denoted in the figure descriptions. No statistical method was used to determine sample size.
Data exclusions	No data were excluded from the datasets.
Replication	All experiments were repeated at least twice with similar results. Microscopic images are representative images of at least three independent experiments and five separate observations within the same experiment. The density profile, molecular cluster, mean squared displacement, and coil interaction analyses are from three simulation replicates. Snapshots are representative of simulations of three independent replicates.
Randomization	No animal or human research participants were used, therefore there was no reason for randomization.
Blinding	Researchers conducting molecular simulations were mostly not aware of in vivo behavior of proteins.

Reporting for specific materials, systems and methods

We require information from authors about some types of materials, experimental systems and methods used in many studies. Here, indicate whether each material, system or method listed is relevant to your study. If you are not sure if a list item applies to your research, read the appropriate section before selecting a response.

Materials & experimental systems

n/a	Involved in the study
☒	☐ Antibodies
☐	☒ Eukaryotic cell lines
☒	☐ Palaeontology and archaeology
☒	☐ Animals and other organisms
☒	☐ Clinical data
☒	☐ Dual use research of concern
☒	☐ Plants

Methods

n/a	Involved in the study
☒	☐ ChIP-seq
☒	☐ Flow cytometry
☒	☐ MRI-based neuroimaging

Eukaryotic cell lines

Policy information about cell lines and Sex and Gender in Research

Cell line source(s)	We used HEK293T (CRL-3216), NIH3T3 (CRL-1658) and Jurkat (TIB-152) cell lines all aquired from ATCC culture collection.
Authentication	The cell lines were authenticated based on morphology comparison with ATCC culture collection database images.
Mycoplasma contamination	The lab regularly tests cell lines in use for mycoplasma infection. No infection on cell lines HEK293T, NIH3T3 or Jurkat has been detected during work presented in this study.
Commonly misidentified lines (See ICLAC register)	No commonly misidentified cell lines were used.

Plants

Seed stocks	*Report on the source of all seed stocks or other plant material used. If applicable, state the seed stock centre and catalogue number. If plant specimens were collected from the field, describe the collection location, date and sampling procedures.*
Novel plant genotypes	*Describe the methods by which all novel plant genotypes were produced. This includes those generated by transgenic approaches, gene editing, chemical/radiation-based mutagenesis and hybridization. For transgenic lines, describe the transformation method, the number of independent lines analyzed and the generation upon which experiments were performed. For gene-edited lines, describe the editor used, the endogenous sequence targeted for editing, the targeting guide RNA sequence (if applicable) and how the editor was applied.*
Authentication	*Describe any authentication procedures for each seed stock used or novel genotype generated. Describe any experiments used to assess the effect of a mutation and, where applicable, how potential secondary effects (e.g. second site T-DNA insertions, mosiacism, off-target gene editing) were examined.*
